# Supplementary material for: Mitochondrial ROS Produced in Human Colon Carcinoma Associated with Cell Survival via Autophagy
Source: Cancers (Basel). 2022 Apr 8;14(8):1883. doi: 10.3390/cancers14081883 (PMC9025374; doi:10.3390/cancers14081883)
Supplement: Supplementary file 1 [file cancers-14-01883-s001.zip › cancers-1650494-supplementary - FileS1-xml.pdf]

Supplementary Information

Materials and Methods

mROS measurement

Mitochondria ROS levels were assessed using red fluorescence mitochondrial superoxide indicator MitoSOX (Invitrogen, Grand Island, NY). Cells were incubated with 5 μM MitoSOX and Hoechst 33342 (Life Technologies, Grand Island, NY) for 10 min, washed with media or 1X PBS three times and fixed with 4% formaldehyde. MitoSOX and Hoechst staining results were analyzed using a Zeiss LSM 980 confocal microscope (Carl Zeiss MicroImaging, Thornwood, NY), and the fluorescence intensity of MitoSOX was measured by using ImageJ software.

Acridine orange stainings

HEK293 and MT cells were grown on 15 mm coverslips at a density of $1.3x10^5$ cells/well in 6-well plates The cells were then treated with drugs for 24 h, and then treated 5 μg/mL AO (Sigma-Aldrich). Nuclei were stained with Hoechst. Then the cells were incubated for 20 min, fixed with 4% PFA, and washed three times with media PBS. Images were obtained using an LSM880 confocal microscope at 400x magnification. Red fluorescence intensity was quantified using Image J2 software.

In Vivo Mouse Tumor Xenograft Assay

Mice were housed in a pathogen-free facility at the Laboratory Animal Research Center of Yonsei University (Seoul, Korea). These mice were handled in accordance with the Institutional Animal Care and Use Committee (permission number: IACUC-A-201901-859-01) and International Guidelines for the Ethical Use of Animals. For intracranial tumor models, 2×10^5 HCT116 cells were implanted into the right hemispheres of 8–10-wk-old male experimental mice via stereotaxic injection. After 1 week, mice were randomly selected and separated into four groups (four mice per group) and intraperitoneally treated with vehicle or A1938 (10 mg/kg) every two days for 16 days. Tumor volume was calculated according to the formula 0.5 × A × B2, where A is the longest diameter of a tumor, and B is its perpendicular diameter. Vehicle and drugs were dissolved in a saline:Ethanol:Tween 80 solution at a ratio of 88:2:18. The tumor volume and mouse body weight were measured daily using the following formula: π/6 × length × width × height. After 8 times of drug treatment, mice were sacrificed, and tissue samples were obtained. The tumors were surgically removed and slowly frozen with dry ice (−70 °C).

Immunoblotting

Soluble proteins were harvested from cells using SDS lysis buffer comprised of 50 mM Tris HCl with a pH of 6.8, containing 10% glycerol, 2% SDS, 10 mM dithiothreitol, and 0.005% bromophenol blue. Equal concentrations of proteins were separated by 11% or 12.5% SDS-PAGE and transferred to polyvinylidene fluoride membranes (EMD Millipore, Billerica, MA, USA). Blots were then blocked and immunolabeled overnight at 4°C with the following primary antibodies: anti-actin (Abcam, ab6276), anti-LC3B (Cell Signaling Technology, 2775), anti-p62 (BD Biosciences). Immunolabeling was visualized using an enhanced chemiluminescence kit (Amersham Life Science, Chalfont, UK) according to the manufacturer's instructions. Images were quantified using Image Lab software (Bio-Rad, Hercules, CA). Actin and GAPDH were used as internal controls. All band intensities were proportional to the amount of target protein on the membrane as determined by the linear range of detection. Images were quantified using Image LabTM software (Bio-Rad).

Statistical analysis

All data are expressed as the means ± SEM with GraphPad Prism (ver. 5.00 for Windows; GraphPad Software, Inc., San Diego, CA). Data were obtained from at least three independent experiments. Statistical analyses were performed using an unpaired, two-tailed Student's *t*-test. P-values less than 0.05 were considered statistically significant (* indicates $p < 0.05$, ** indicates $p < 0.01$, *** indicates $p < 0.001$).
