# Supplementary material for: Mitochondrial ROS Produced in Human Colon Carcinoma Associated with Cell Survival via Autophagy
Source: Cancers (Basel). 2022 Apr 8;14(8):1883. doi: 10.3390/cancers14081883 (PMC9025374; doi:10.3390/cancers14081883)
Supplement: Supplementary file 1 [file cancers-14-01883-s001.zip › cancers-1650494-supplementary Figure-xml.pdf]

**Supplementary information**

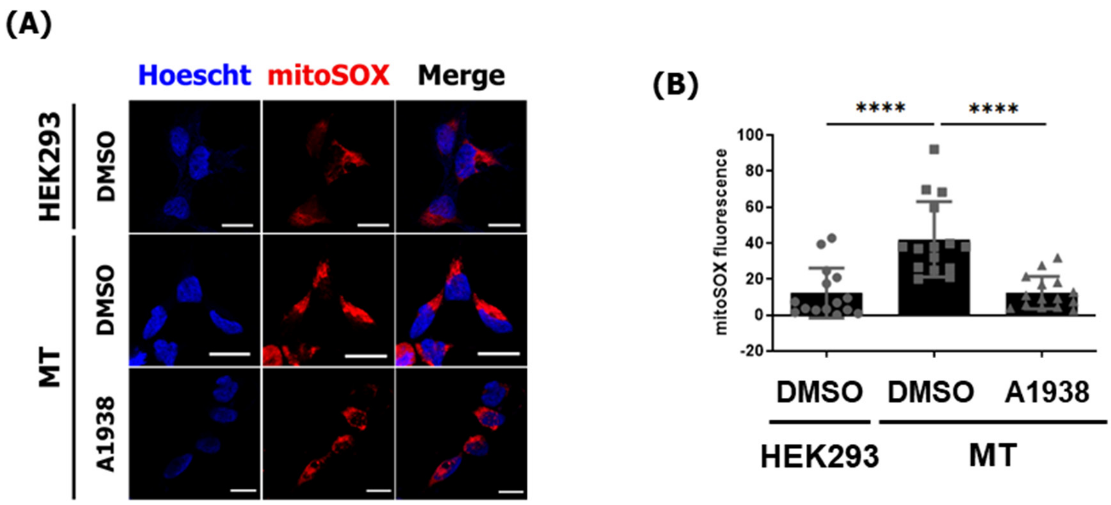


**Figure S1.** The effect of A1938 on mROS generation. (A) Confocal microscopy images of the MitoSOX Red fluorescence of mROS generated in HEK293 and MT cells. A1938, 30 µM. 4 h treatment. Scale bar, 20 µm. (B) Graph shows mean ± SEM of cells (n = 15). ns, ****p < 0.001.

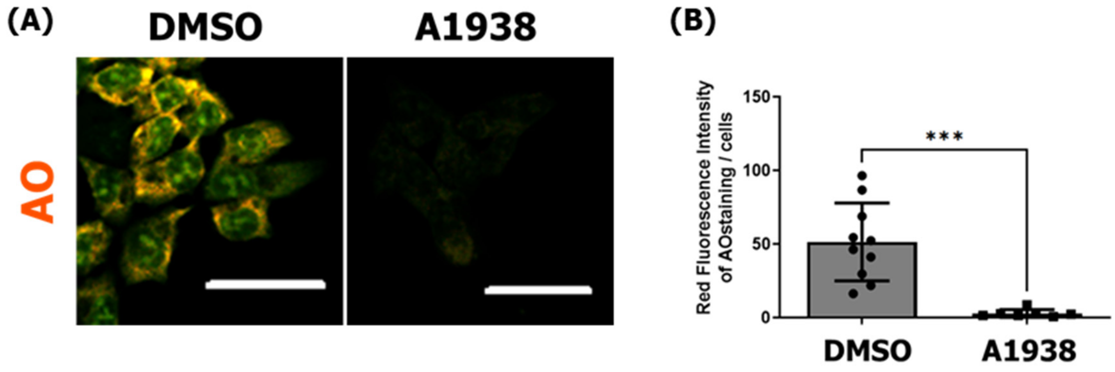


**Figure S2.** A1938 treatment on lysosomal activity in HCT116 cells. (A) HCT116 cells were treated with DMSO control or A1938 30 µM for 24 h. Live cells were stained with AO 2 µg/mL for 25 min, fixed, and examined by confocal fluorescence microscopy. Confocal fluorescence microscopy images of HCT116 cells treated with A1938 in serum free RPMI media. (B) Average AO intensity per cell of HCT116 cells treated with A1938 in serum free RPMI media (n > 10). Scale bar, 20 µm. ***P < 0.001.

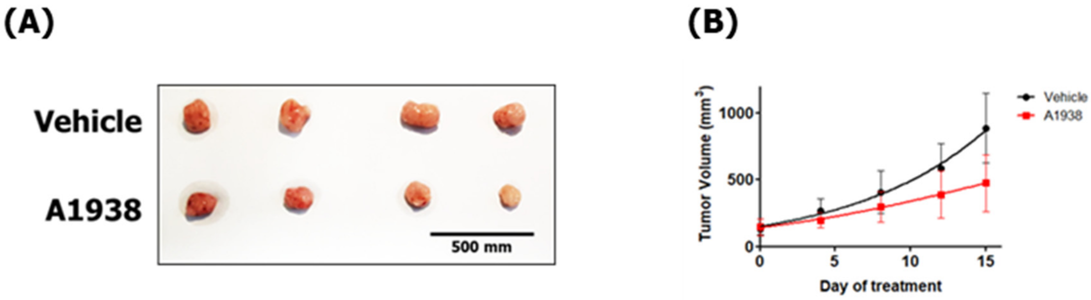


**Figure S3.** UQCRB inhibitor A1938 attenuated HCT116 xenograft tumor growth. (A) Representative images of tumors extracted from nude mice bearing HCT116 cells in different groups. (B) Mouse tumor volumes (n = 4/group).

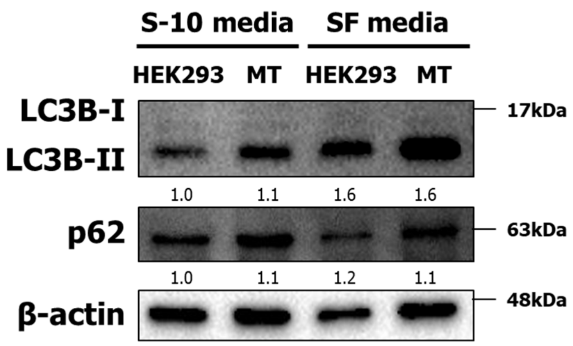


**Figure S4.** Autophagy marker protein level in the presence or absence serum. HEK293 and MT cells treated with either 10% serum-containing medium or serum-free medium for 4 h and then analyzed by western blot analysis using antibodies against LC3B and p62. Relative band intensities value was normalized to β-actin (loading control). The normalized protein levels are shown under each band.

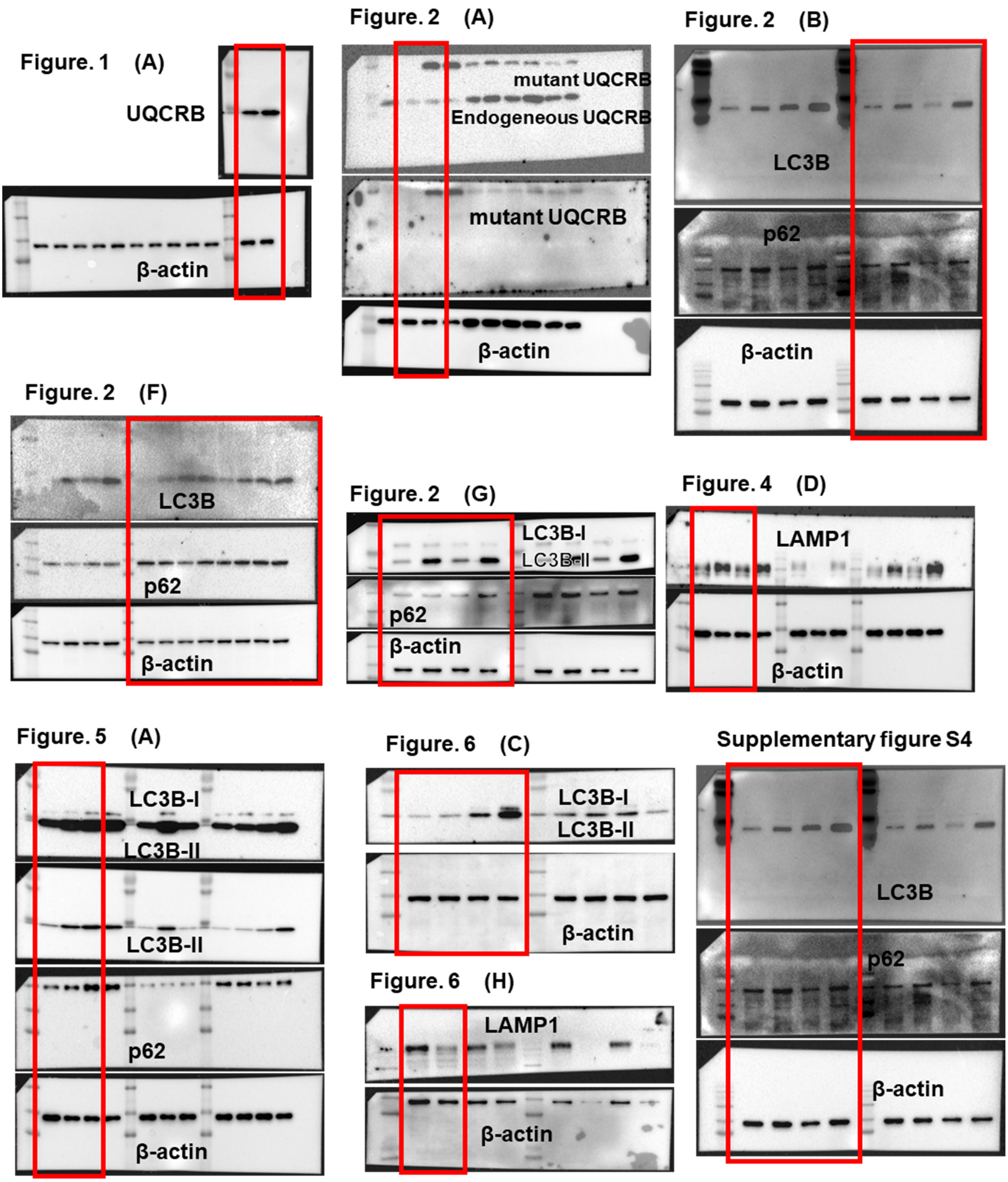


**Figure S5.** Original western blots
